# Association of CAR-T approval on outcomes in patients with diffuse large B-cell lymphoma at the population level in the United States

**DOI:** 10.1186/s40364-025-00780-4

**Published:** 2025-04-24

**Authors:** John L. Vaughn, Angela Ramdhanny, Malak Munir, Sravani Rimmalapudi, Narendranath Epperla

**Affiliations:** 1 Division of Hematology & Oncology, NYU Grossman Long Island School of Medicine, New York, NY USA; 2 Karsh Division of Gastroenterology and Hepatology, Cedars-Sinai Medical Center, Los Angeles, CA USA; 3 Division of Hematology and Hematologic Malignancies, Huntsman Cancer Institute, University of Utah, Salt Lake City, UT USA; 4 Division of Hematology and Hematologic Malignancies, Department of Medicine, Huntsman Cancer Institute, Salt Lake City, UT 84103 USA

**Keywords:** Diffuse large B-cell lymphoma, DLBCL, CAR-T, Survival, Outcomes

## Abstract

While the advent of chimeric antigen receptor T-cell (CAR-T) therapy has revolutionized the treatment of relapsed or refractory DLBCL, it is unclear how survival has changed at the population level following its approval. Herein, we performed a population-based cohort study using the SEER-17 database. The primary exposure was a period of diagnosis (2014–2017 vs. 2018–2021), and these periods were selected based on the first FDA-approval of CAR-T in 2017. Study outcomes were relative survival (RS), overall survival (OS), lymphoma-specific survival (LSS), and the cumulative incidence of death from lymphoma (CIF). A total of 51,584 patients with DLBCL were included in the study with 24,861 patients diagnosed in time period-1 (2014–2017) and 26,723 patients diagnosed in time period-2 (2018–2021). The median age at diagnosis was 68 years (interquartile range, 57–77) and most patients were White (*n* = 42,190, 82%) with advanced stage at diagnosis (*n* = 28,203, 55%). In unadjusted analysis, the 5-year RS (95% CI) increased from 64% from 2014 to 2017 to 66% from 2018 to 2021, while 5-year OS increased from 54 to 55%, and 5-year LSS increased from 64 to 66%. On competing risks analysis, the 5-year probability of death from lymphoma decreased from 34 to 31%. The improvements in survival were observed across age, disease stage, and racial groups, and remained significant when adjusting for age, sex, race, stage, B symptoms and documented receipt of chemotherapy in multivariable survival models (adjusted OS HR = 0.97, 95%CI = 0.94-1.00, *p* = 0.04; adjusted LSS HR = 0.93, 95%CI = 0.90–0.96, *p* < 0.001). We found improved survival for patients with DLBCL diagnosed between 2018 and 2021 when compared to those diagnosed between 2014 and 2017. These findings will serve as the benchmark for future studies evaluating the impact of CAR-T administered earlier in their disease course.

**Supplementary Information:**

The online version contains supplementary material available at 10.1186/s40364-025-00780-4.

To the Editor,

Diffuse large B-cell lymphoma (DLBCL) is curable in approximately 60% of patients with the current first-line anthracycline based chemoimmunotherapy^1^. However, 10–15% of patients will have primary refractory disease and 25–30% of patients will experience relapse 2, 3. The subgroup of patients with primary refractory DLBCL or early first relapse have a particularly poor prognosis 4, 5. The advent of chimeric antigen receptor T-cell (CAR-T) therapy has revolutionized the treatment of relapsed/refractory DLBCL. In the multicenter phase 2 ZUMA-1 trial, axicabtagene ciloleucel had an objective response rate of 82% with a complete response rate of 54%. 6 The overall survival (OS) at 18 months was 52%. This led to the first US FDA approval of CAR-T for DLBCL in 2017. Similar results were subsequently observed in real-world studies.^7^ Since then, the number of CAR-T cell infusions administered to patients in the US for DLBCL has increased rapidly each year to 1,747 infusions in 2022.^8^ However, it is unclear how survival has changed at the population level following the commercial introduction of CAR-T. Hence, we performed a population-based cohort study using SEER-17 database to evaluate this.

We included patients aged 18 years and older with pathologically confirmed DLBCL diagnosed in USA between 2014 and 2021. The study population was divided into two time periods, time period-1 (2014–2017) and time period-2 (2018–2021). The time periods were selected based on the first FDA-approval of CAR-T therapy for DLBCL (which was Oct. 2017). Patients who were diagnosed by autopsy or death certificate or who had primary central nervous system lymphoma were excluded.

The study outcomes were relative survival (RS), OS, lymphoma-specific survival (LSS), and the cumulative incidence of death from lymphoma (CIF). The primary exposure was year of diagnosis (period-1 versus period-2). The study covariates were age, sex, race (White, Black, and Other), stage, documentation of B symptoms, and documentation of whether a patient received chemotherapy for their lymphoma. These variables were selected for incorporation into our multivariable models on conceptual grounds since they have all been found to predict survival in lymphoma patients under different circumstances. Additional details of the statistical analysis are presented in Table S1.

51,584 patients with DLBCL were included in the study with 24,861 patients diagnosed in time period-1 (2014–2017) and 26,723 patients in time period-2 (2018–2021). The median age at diagnosis was 68 years [interquartile range (IQR), 57–77]. Most patients were White (*n* = 42,190, 82%) and had advanced stage at diagnosis (*n* = 28,203, 55%). The presence of B symptoms was documented in 26% (*n* = 13,529) of patients. Most patients (*n* = 40,352, 78%) had documented chemotherapy receipt with a median time from diagnosis to treatment of 20 days (IQR, 7–35). Patient characteristics were comparable between two time periods (Table 1). Median follow-up was 5.75 years (95% CI, 5.75–5.83) for time period-1 and 1.83 years (95% CI, 1.83–1.91) for time period-2.

**Table 1:** Patient characteristics

	Total	2014–2017	2018–2021
	*N* = 51,584	*N* = 24,861	*N* = 26,723
Median age at diagnosis, y (IQR)	68 (57–77)	68 (57–77)	68 (58–77)
Age group			
18–44	4,828 (9%)	2,350 (9%)	2,478 (9%)
45–54	5,258 (10%)	2,733 (11%)	2,525 (9%)
55–64	10,194 (20%)	4,994 (20%)	5,200 (19%)
65–74	14,029 (27%)	6,539 (26%)	7,490 (28%)
75–89	15,526 (30%)	7,441 (30%)	8,085 (30%)
Missing	1,749 (3%)	804 (3%)	945 (4%)
Sex			
Female	22,619 (44%)	10,847 (44%)	11,772 (44%)
Male	28,965 (56%)	14,014 (56%)	14,951 (56%)
Race			
White	42,190 (82%)	20,467 (82%)	21,723 (81%)
Black	3,592 (7%)	1,758 (7%)	1,834 (7%)
Other	5,319 (10%)	2,463 (10%)	2,856 (11%)
Missing	483 (1%)	173 (1%)	310 (1%)
Ann Arbor stage			
Stage I-II	19,983 (39%)	9,795 (39%)	10,188 (38%)
Stage III-IV	28,203 (55%)	13,259 (53%)	14,944 (56%)
Missing	3,398 (7%)	1,807 (7%)	1,591 (6%)
B symptoms			
No B symptoms	28,692 (56%)	13,959 (56%)	14,733 (55%)
B symptoms	13,524 (26%)	6,734 (27%)	6,790 (25%)
Missing	9,368 (18%)	4,168 (17%)	5,200 (19%)
Chemotherapy			
No/Unknown	11,251 (22%)	5,404 (22%)	5,847 (22%)
Yes	40,333 (78%)	19,457 (78%)	20,876 (78%)
Median time to treatment, days	20 (7–35)	19 (7–34)	21 (8–36)

Abbreviations: IQR = Interquartile range

Among patients with DLBCL diagnosed in the US and reported to SEER cancer registries, the 5-year RS (95% CI) increased from 64% (64–65%) between 2014 and 2017 to 66% (65–67%) between 2018 and 2021. Five-year OS increased from 54% (53–54%) to 55% (54–56%). Five-year LSS increased from 64% (64–65%) to 66% (65–67%). On competing risks analysis, the 5-year probability of death from lymphoma decreased from 34% (34–35%) to 31% (30–31%). The restricted mean survival times are shown in Table S2.

We also performed a subgroup analysis that restricted the survival estimates to only those patients with a documented history of chemotherapy (*n* = 40,333,78%) and the results were consistent with the original analysis (Table S3).

We next examined the outcomes of patients by age, stage, and race. Among patients less than 65 years of age at the time of diagnosis, 5-year RS (95% CI) increased from 76% (75–76%) between 2014 and 2017 to 77% (76–78%) between 2018 and 2021. Among older patients, 5-year RS increased from 54% (53–55%) to 57% (55–58%). Additional data by age are shown in Tables S4 and S5. The RS curves for each period and age at diagnosis are shown in Figure S1. We then analyzed outcomes by stage. For patients with limited stage disease, 5-year RS increased from 77% (76–77%) to 78% (77–79%). For patients with advanced stage disease, 5-year RS increased from 57% (56–58%) to 58% (57–60%). Table S6 shows the results for the other study outcomes by stage. The RS curves for each period and stage are shown in Figure S2. As shown in Table S7, the improvements were consistent across racial groups as well.

For each outcome, we constructed univariable and multivariable flexible parametric survival models to test for improvements between time periods while adjusting for differences in age, sex, race, stage, documented B symptoms, and documented receipt of chemotherapy. The unadjusted and adjusted hazard ratios are shown in Table 2. There was a small but statistically significant improvement in all the outcomes across study periods. The largest improvement was seen in the competing risks model where there was a 14% improvement in the sub-distribution hazard rate for death from lymphoma [subhazard ratio 0.86; 95% CI, 0.83–0.89; *P* < 0.001]. The smallest improvement was seen for OS where there was only a 3% improvement between time periods [HR 0.97; 95% CI, 0.94-1.00; *P* = 0.04).

**Table 2:** Univariable and multivariable flexible parametric survival models

Outcome	Unadjusted HR (95% CI)	*p*-value	Adjusted HR (95% CI)*	*p*-value
RS				
2014–2017	Reference		Reference	
2018–2021	0.97 (0.94–1.01)	0.12	0.96 (0.92–0.99)	0.01
OS				
2014–2017	Reference		Reference	
2018–2021	0.99 (0.96–1.01)	0.33	0.97 (0.94-1.00)	0.047
LSS				
2014–2017	Reference		Reference	
2018–2021	0.95 (0.92–0.98)	0.002	0.93 (0.90–0.96)	< 0.001
CIF				
2014–2017	Reference		Reference	
2018–2021	0.92 (0.89–0.95)	< 0.001	0.86 (0.83–0.89)	< 0.001

Abbreviations: CI = confidence interval, CIF = cumulative incidence of death from lymphoma, RS = relative survival, OS = overall survival, LSS = lymphoma-specific survival

*Models were adjusted for age, sex, race, stage, documented presence of B-symptoms at diagnosis, and documented receipt of chemotherapy

CAR-T has shown significant activity in relapsed/refractory disease and has been widely utilized since 2017, however, the translation of survival improvement to the population level is unclear. In this comprehensive analysis using the SEER-17 registry, we report improvements in several outcomes for patients diagnosed during the post-CAR-T period (2018–2021) compared to pre-CAR-T (2014–2017). We found a 4% improvement in the hazard rate for relative survival, a 3% improvement in the hazard rate for overall survival, a 7% improvement in the hazard rate for lymphoma-specific survival, and a 14% improvement in the sub-distribution hazard rate for death from lymphoma (taking into consideration the competing risk of death from other causes). These improvements were observed across age, disease stage, and racial groups.

The gap between clinical trial/real-world estimates and our population-level outcomes could reflect several limiting factors for CAR-T utilization in practice on a national level 9. Selection bias in real-world analyses from major cancer centers does not account for disparities in healthcare access across the US. Such limitations can impact national accessibility to CAR-T. Furthermore, manufacturing challenges, including production failures, outsourcing requirements to specialized biopharmaceutical facilities, and prolonged manufacturing times pose barriers to treatment 10. These limitations were further augmented by the COVID-19 pandemic, potentially attenuating the observable survival benefit at the population level during our study period 11, 12.

Logistical limitations also present additional challenges to CAR-T accessibility. These requirements can create barriers for eligible patients who reside far from treatment centers 13. Furthermore, socioeconomic disparities in healthcare access are magnified in the context of CAR-T therapy, with studies demonstrating lower utilization rates among racial minorities 14. The substantial cost of CAR-T, exceeding $350,000 per patient, likely exacerbates these disparities 15. Our findings show a significant but modest improvement in survival at the population level for patients with DLBCL during the post-CAR-T era despite limited utilization and the presence of barriers to CAR-T during our study period. Nonetheless, survival outcomes of DLBCL are projected to improve with the recent studies evaluating CAR-T in the first-line setting, as well as the introduction of novel therapies such as polatuzumab vedotin and bispecific T-cell engagers.

The limitations of our study include a lack of data regarding specific treatment regimens, prior lines of therapy, comorbidities, and performance status since these data are not reported to cancer registries in the United States. The lack of these data prevents us from restricting our analysis only to patients with relapsed/refractory DLBCL and adjusting for potential confounders. Additionally, the SEER database may not be fully representative of the entire US population, and follow-up was limited for patients diagnosed during the most recent time period. These limitations may have biased our results, possibly leading to an underestimation or overestimation of the therapeutic impact of CAR-T cell therapy on the US population. Future studies could benefit by incorporating data from health insurance claims to identify specific treatment regimens and additional covariates. The SEER database is linked with Medicare claims every 2 years, which may facilitate additional real-world studies in patients over the age of 65 years.

This is the first study to demonstrate improved survival for patients with DLBCL at the population level who were diagnosed between 2018 and 2021 when compared to those diagnosed between 2014 and 2017 and corresponds to CAR-T therapy approval in the US. In addition to CAR-T cell therapy, the approval of other therapies may have contributed to our findings including polatuzumab vedotin (in combination with bendamustine and rituximab) in 2019 and tafasitamab-cxix in 2020. The small improvement in survival likely reflects the limited use of new therapies in a subset of patients with relapsed/refractory disease and the challenging circumstances surrounding COVID-19 pandemic causing decreased access to new therapies. These findings will serve as the benchmark for future studies evaluating the impact of CAR-T, especially administered earlier in their disease course.

## Electronic supplementary material

Below is the link to the electronic supplementary material.

Supplementary Material 1

## Data Availability

No datasets were generated or analysed during the current study.
