# Supplementary material for: Association of CAR-T approval on outcomes in patients with diffuse large B-cell lymphoma at the population level in the United States
Source: Biomark Res. 2025 Apr 24;13:64. doi: 10.1186/s40364-025-00780-4 (PMC12023351; doi:10.1186/s40364-025-00780-4)

**SUPPLEMENTAL APPENDIX**

**Table of Contents** 1

**Definitions and Statistical Analysis:** Table S1 2

**Restricted Mean Survival Times:** Table S2 3

**Outcomes for Patients with a Known History of Chemotherapy:** Table S3 4

**Outcomes by Age:** Table S4 5

**Outcomes for Patients Aged 75-89 Years:** Table S5 6

**Outcomes by Stage:** Table S6 7

**Outcomes by Race:** Table S7 8

**RS Curves by Age:** Figure S1 9

**RS Curves by Stage:** Figure S2 10

**Table S1. Definitions and Statistical Analysis**

| **Definitions**  RS was defined as the ratio of all-cause survival to expected survival in a comparable group of individuals from the general population. We estimated expected survival by matching patients in our study to individuals in the general population by age, sex, year, and race using data provided by SEER. OS was defined as the probability of death from any cause following diagnosis of lymphoma. LSS was defined as the probability of survival when lymphoma was considered the only possible cause of death. The CIF was defined as the crude probability of death from lymphoma considering the competing risk of death from other causes. |
| --- |
| **Statistical Analysis**  We analyzed patient characteristics using descriptive statistics. Median follow-up times were estimated using the Reverse Kaplan-Meier method. Unadjusted restricted mean survival times were estimated up to a maximum time of 3.91 years, corresponding to the maximum follow-up time for patients diagnosed between 2018-2021. Unadjusted 5-year estimates for each study outcome were obtained from flexible parametric survival models using 5 degrees of freedom (6 knots) of freedom for the baseline log cumulative hazard. We tested the hypothesis that RS was significantly higher following the approval of CAR-T therapy using a multivariable flexible parametric survival model with 5 degrees of freedom (6 knots) for the baseline log cumulative hazard.  For all the models in this study, the period of diagnosis variable was modeled as a time-dependent variable with 2 degrees of freedom (3 knots) to relax the proportional hazards assumption. All the study covariates were included in the final multivariable model. We tested for significant interactions between the primary exposure and each covariate in a stepwise fashion using the likelihood ratio test. Missing data were handled using multiple imputation with chained equations with 20 imputations based on the frequency of missing data. P-values less than 0.05 were considered significant. Analyses were performed using STATA version 18.5. The flexible parametric models were created using the *stpm2* and *stcrprep* packages. |

**Table S2. Restricted Mean Survival Times in Years (95% CI)**

| **Outcome** | **2014-2017** | **2018-2021** |
| --- | --- | --- |
| RS | 2.81 (2.79-2.83) | 2.84 (2.82-2.87) |
| OS | 2.63 (2.61-2.65) | 2.65 (2.63-2.67) |
| LSS | 2.85 (2.83-2.87) | 2.90 (2.88-2.92) |

**Abbreviations:** LSS = lymphoma-specific survival, OS = overall survival, RS = relative survival

**Table S3. Outcomes for Patients with a Known History of Chemotherapy**

| **Outcome** | **2014-2017** | **2018-2021** |
| --- | --- | --- |
| 5-year RS (95% CI) | 0.72 (0.71-0.73) | 0.73 (0.72-0.74) |
| 5-year OS (95% CI) | 0.62 (0.61-0.63) | 0.62 (0.61-0.63) |
| 5-year LSS (95% CI) | 0.71 (0.70-0.72) | 0.73 (0.72-0.74) |
| 5-year CIF (95% CI) | 0.28 (0.28-0.29) | 0.25 (0.24-0.26) |

**Abbreviations:** CI = confidence interval, CIF = cumulative incidence of death from lymphoma, LSS = lymphoma-specific survival, OS = overall survival, RS = relative survival

**Table S4. Outcomes by Age**

| **Outcome** | **2014-2017** | **2018-2021** |
| --- | --- | --- |
| 5-year RS (95% CI) |  |  |
| < 65 years | 0.76 (0.75-0.76) | 0.77 (0.76-0.78) |
| ≥ 65 years | 0.54 (0.53-0.55) | 0.57 (0.55-0.58) |
| 5-year OS (95% CI) |  |  |
| < 65 years | 0.72 (0.71-0.72) | 0.73 (0.72-0.74) |
| ≥ 65 years | 0.42 (0.41-0.43) | 0.44 (0.42-0.45) |
| 5-year LSS (95% CI) |  |  |
| < 65 years | 0.77 (0.77-0.78) | 0.79 (0.78-0.80) |
| ≥ 65 years | 0.55 (0.54-0.56) | 0.58 (0.57-0.59) |
| 5-year CIF (95% CI) |  |  |
| < 65 years | 0.22 (0.21-0.23) | 0.19 (0.18-0.20) |
| ≥ 65 years | 0.42 (0.42-0.43) | 0.38 (0.37-0.38) |

**Abbreviations:** CI = confidence interval, CIF = cumulative incidence of death from lymphoma, LSS = lymphoma-specific survival, OS = overall survival, RS = relative survival

**Table S5. Outcomes for Patients Aged 75-89 Years**

| **Outcome** | **2014-2017** | **2018-2021** |
| --- | --- | --- |
| 5-year RS (95% CI) | 0.45 (0.44-0.46) | 0.47 (0.46-0.49) |
| 5-year OS (95% CI) | 0.31 (0.30-0.32) | 0.33 (0.31-0.34) |
| 5-year LSS (95% CI) | 0.45 (0.44-0.46) | 0.48 (0.47-0.49) |
| 5-year CIF (95% CI) | 0.50 (0.50-0.51) | 0.45 (0.44-0.46) |

**Abbreviations:** CI = confidence interval, CIF = cumulative incidence of death from lymphoma, LSS = lymphoma-specific survival, OS = overall survival, RS = relative survival

**Table S6. Outcomes by Stage**

| **Outcome** | **2014-2017** | **2018-2021** |
| --- | --- | --- |
| 5-year RS (95% CI) |  |  |
| Limited | 0.77 (0.76-0.77) | 0.78 (0.77-0.79) |
| Advanced | 0.57 (0.56-0.58) | 0.58 (0.57-0.60) |
| 5-year OS (95% CI) |  |  |
| Limited | 0.64 (0.63-0.65) | 0.65 (0.64-0.66) |
| Advanced | 0.47 (0.47-0.48) | 0.48 (0.47-0.50) |
| 5-year LSS (95% CI) |  |  |
| Limited | 0.75 (0.75-0.76) | 0.77 (0.76-0.78) |
| Advanced | 0.57 (0.56-0.58) | 0.59 (0.58-0.61) |
| 5-year CIF (95% CI) |  |  |
| Limited | 0.24 (0.23-0.24) | 0.21 (0.20-0.22) |
| Advanced | 0.41 (0.40-0.42) | 0.37 (0.36-0.38) |

**Abbreviations:** CI = confidence interval, CIF = cumulative incidence of death from lymphoma, LSS = lymphoma-specific survival, OS = overall survival, RS = relative survival

**Table S7. Outcomes by Race**

| **Outcome** | **2014-2017** | **2018-2021** |
| --- | --- | --- |
| 5-year RS (95% CI) |  |  |
| White | 0.65 (0.64-0.65) | 0.66 (0.65-0.67) |
| Black | 0.61 (0.59-0.63) | 0.63 (0.61-0.65) |
| Other | 0.63 (0.61-0.64) | 0.64 (0.62-0.66) |
| 5-year OS (95% CI) |  |  |
| White | 0.54 (0.53-0.54) | 0.54 (0.53-0.55) |
| Black | 0.53 (0.51-0.54) | 0.53 (0.51-0.55) |
| Other | 0.54 (0.53-0.56) | 0.55 (0.53-0.57) |
| 5-year LSS (95% CI) |  |  |
| White | 0.64 (0.64-0.65) | 0.66 (0.65-0.67) |
| Black | 0.63 (0.61-0.65) | 0.65 (0.63-0.67) |
| Other | 0.64 (0.63-0.65) | 0.66 (0.64-0.67) |
| 5-year CIF (95% CI) |  |  |
| White | 0.34 (0.34-0.35) | 0.31 (0.30-0.32) |
| Black | 0.35 (0.34-0.37) | 0.32 (0.30-0.34) |
| Other | 0.34 (0.33-0.36) | 0.31 (0.30-0.32) |

**Abbreviations:** CI = confidence interval, CIF = cumulative incidence of death from lymphoma, LSS = lymphoma-specific survival, OS = overall survival, RS = relative survival

**Figure S1. (A) RS for patients < 65 years of age. (B) RS for patients ≥ 65 years of age.**


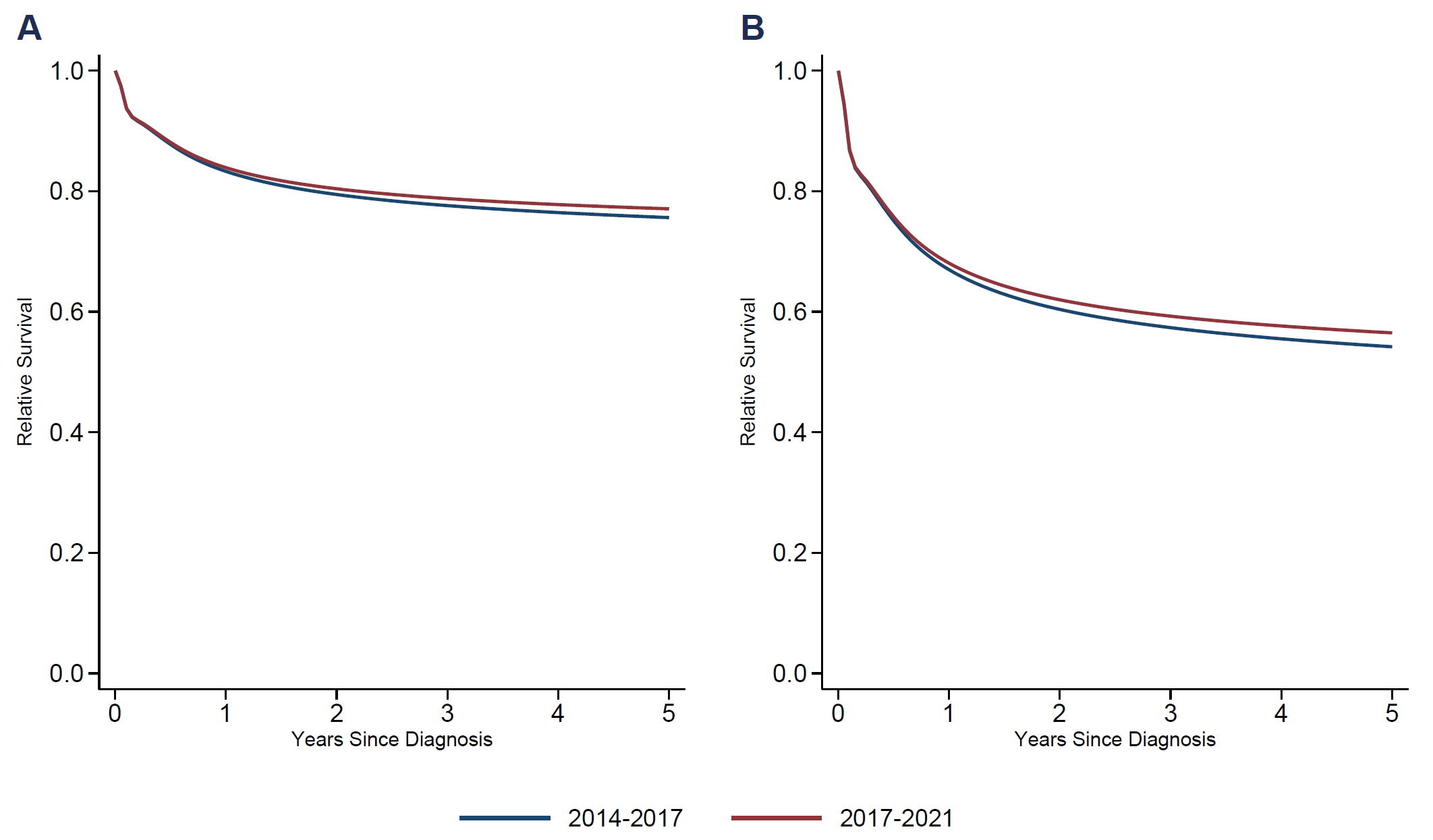


**Figure S2. (A) RS for patients with limited stage disease. (B) RS for patients with advanced stage disease.**


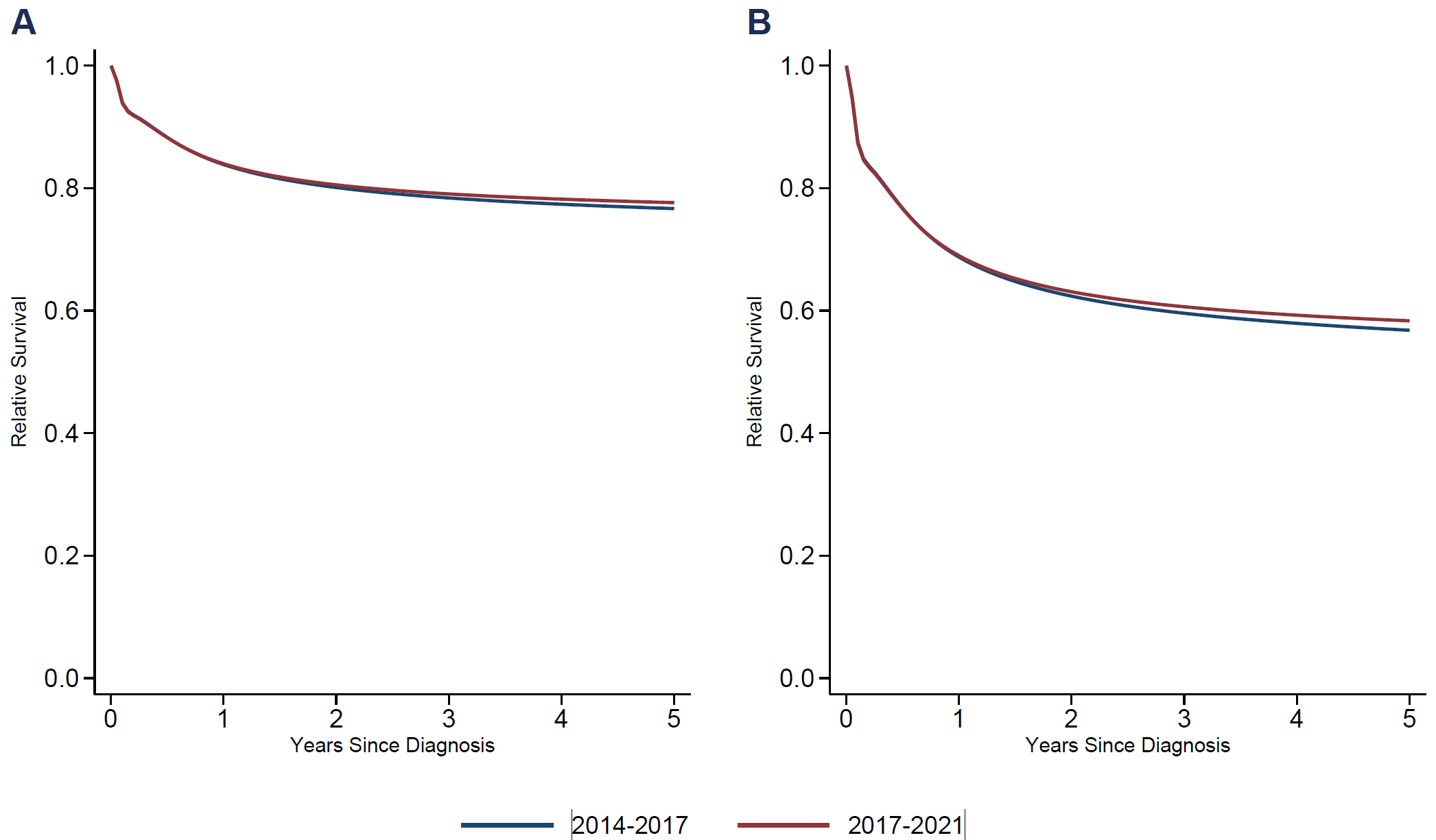

Supplement: Supplementary file 1 — Supplementary Material 1 [file 40364_2025_780_MOESM1_ESM.docx]
